# Advancing donor management research: design and implementation of a large, randomized, placebo-controlled trial

**DOI:** 10.1186/2110-5820-1-20

**Published:** 2011-06-14

**Authors:** Lorraine B Ware, Tatsuki Koyama, Dean Billheimer, Megan Landeck, Elizabeth Johnson, Sandra Brady, Gordon R Bernard, Michael A Matthay

**Affiliations:** 1 Division of Allergy, Pulmonary and Critical Care Medicine, Department of Medicine, Vanderbilt University, Nashville, TN, USA; 2 Department of Biostatistics, Vanderbilt University, Nashville, TN, USA; 3 BIO5 Institute, the University of Arizona, Tucson, AZ, USA; 4 California Transplant Donor Network, Oakland, CA, USA; 5 Department of Medicine, University of California, San Francisco, CA, USA; 6 Department of Anesthesia, University of California, San Francisco, CA, USA; 7 Cardiovascular Research Institute, University of California, San Francisco, CA, USA

## Abstract

**Background:**

Given the persistent shortage of organs for transplantation, new donor management strategies to improve both organ utilization and quality of procured organs are needed. Current management protocols for the care of the deceased donor before organ procurement are based on physiological rationale, experiential reasoning, and retrospective studies without rigorous testing. Although many factors contribute to the lack of controlled clinical trials in donor management, a major factor is the unique challenges posed by research in the brain-dead organ donor.

**Methods and Results:**

This article describes the study design and the challenges faced during implementation of the Beta-agonists for Oxygenation in Lung Donors (BOLD) study, a randomized, placebo-controlled clinical trial of nebulized albuterol vs. placebo in 500 organ donors. The study design and implementation are described with emphasis on aspects of the study that are unique to research in brain-dead organ donors.

**Conclusions:**

Experience gained during the design and implementation of the BOLD study should be useful for investigators planning future clinical trials in the brain-dead donor population and for intensivists who are involved in the care of the brain-dead organ donor.

## Introduction

Despite recent efforts to improve donation awareness, family consent, clinical management, and organ utilization, there remains a persistent shortage of organs for transplantation [1] and a plateau in the number of organ donors has been noted. Thus, new strategies to improve the quality of donated organs and rates of organ utilization are still needed. An important strategy to improve organ utilization is through novel donor management therapies that are designed to optimize organ function in the deceased donor, thus maximizing the likelihood of organ utilization and minimizing the likelihood of graft dysfunction.

New donor management therapies should be rigorously evaluated before clinical implementation. Randomized, controlled, clinical trials are the primary route for testing of new pharmacologic therapies and other clinical interventions in living patients. However, there have been very few randomized, clinical trials in deceased donor management [2]. Current management protocols for the care of the organ donor before organ procurement are based on physiological rationale, experiential reasoning, and retrospective studies without the benefit of rigorous testing [3]. Although there are many factors that contribute to the lack of controlled clinical trials in donor management, a major factor is the unique challenges posed by research in the brain-dead organ donor.

To advance the field of donor management and optimize organ utilization, there is a pressing need to apply the science of clinical trial design to the implementation of donor management studies. The purpose of this article is to describe the study design and the challenges faced during implementation of the Beta-agonists for Oxygenation in Lung Donors (BOLD) (NCT #00310401) study, a trial of nebulized albuterol vs. placebo in 500 organ donors. We have placed particular emphasis on aspects that are unique to conducting research in brain-dead organ donors. Experience gained during the design and implementation of the BOLD study should be useful for investigators planning future clinical trials in the brain-dead donor population.

### Study rationale

The demand for lung transplantation exceeds the supply of donor lungs, leading to protracted waiting times and a high death rate on the waiting list [4-6]. Although the use of extended donors who do not meet traditional criteria for lung donation has improved donor lung utilization rates at selected centers [7-10], the national donor lung utilization rate remains low [4]. The most common reasons for failure to utilize donor lungs are donor hypoxemia and/or pulmonary infiltrates [4]. Acute pulmonary edema occurs commonly in association with acute brain injury [4] and is a potentially reversible cause of donor hypoxemia and pulmonary infiltrates. In lungs from 29 donors that were rejected for transplantation, lung wet-to-dry weight ratio, a measure of pulmonary edema, was normal in only 7 (24%), indicating that pulmonary edema is very common in organ donors [11]. Strategies to enhance the resolution of pulmonary edema could lead to improved donor oxygenation and higher rates of donor lung utilization.

The clearance of pulmonary edema fluid from the distal airspaces is driven by active transport of sodium across the alveolar epithelium [12]. Faster rates of alveolar fluid clearance are associated with more rapid improvements in oxygenation in patients with hydrostatic pulmonary edema [13] and better oxygenation, a shorter duration of mechanical ventilation, and improved survival in patients with acute lung injury [14,15]. In addition, in recipients with primary graft dysfunction and reperfusion pulmonary edema after lung transplantation, those with intact alveolar fluid clearance had more rapid improvements in oxygenation than recipients with impaired fluid clearance [16]. Thus, the capacity to resolve alveolar edema is an important variable in determining clinical outcomes across a wide variety of critically ill patients with acute pulmonary edema.

Inhaled beta-2 agonists increase the rate of alveolar fluid clearance and reduce pulmonary edema in both animal and human lungs [12]. In donor lungs that were excised but not transplanted, the majority responded to beta-2 adrenergic agonists instilled into the airspaces with increased rates of alveolar fluid clearance [11,17]. Standard doses of inhaled beta-2 agonists reach concentrations in the pulmonary edema fluid that are sufficient to stimulate alveolar fluid clearance [18]. Based on this evidence, we hypothesized that pharmacologic treatment with an inhaled beta-2 adrenergic agonist to enhance clearance of pulmonary edema from the distal airspaces would reduce donor hypoxemia and increase donor lung utilization. To test this hypothesis, we designed a prospective, randomized, clinical trial to test the efficacy of inhaled albuterol to increase the rate of alveolar fluid clearance and reduce pulmonary edema in brain-dead organ donors.

### Study overview

**Figure 1:**
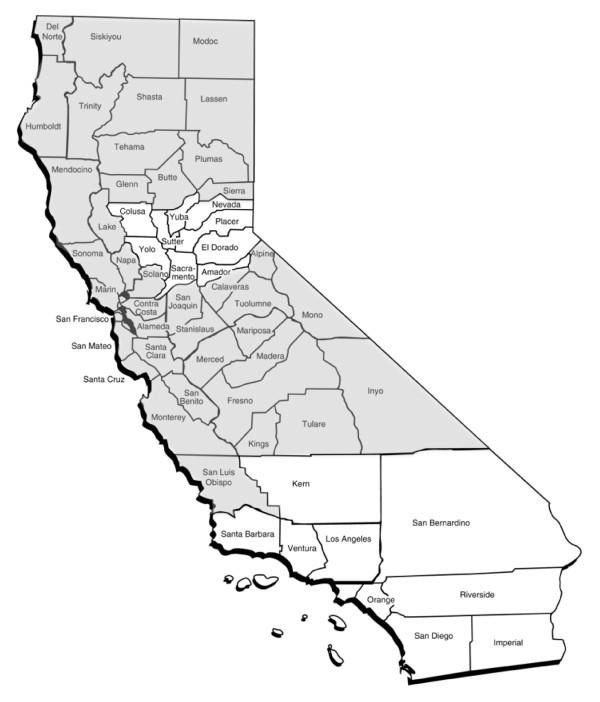
**Geographic area in Northern California served by the California Transplant Donor Network (shaded area)**. Northern Nevada also is served by the California Transplant Donor Network.

The BOLD study is a multicenter, randomized, double-blind, placebo-controlled trial that compared the effects of nebulized albuterol to placebo on donor oxygenation in 500 brain-dead organ donors. The coordinating center for the trial is at Vanderbilt University. Donors are enrolled at 175 hospitals served by the California Transplant Donor Network (CTDN), an organ procurement organization that serves a population of more than 10 million people in Northern California and parts of Nevada (Figure 1). The trial is funded by the National Institutes of Health through the National Heart Lung and Blood Institute and enrolled its first donor in April 2007.

### Clinical and physiological endpoints

#### Primary outcome

**Figure 2:**
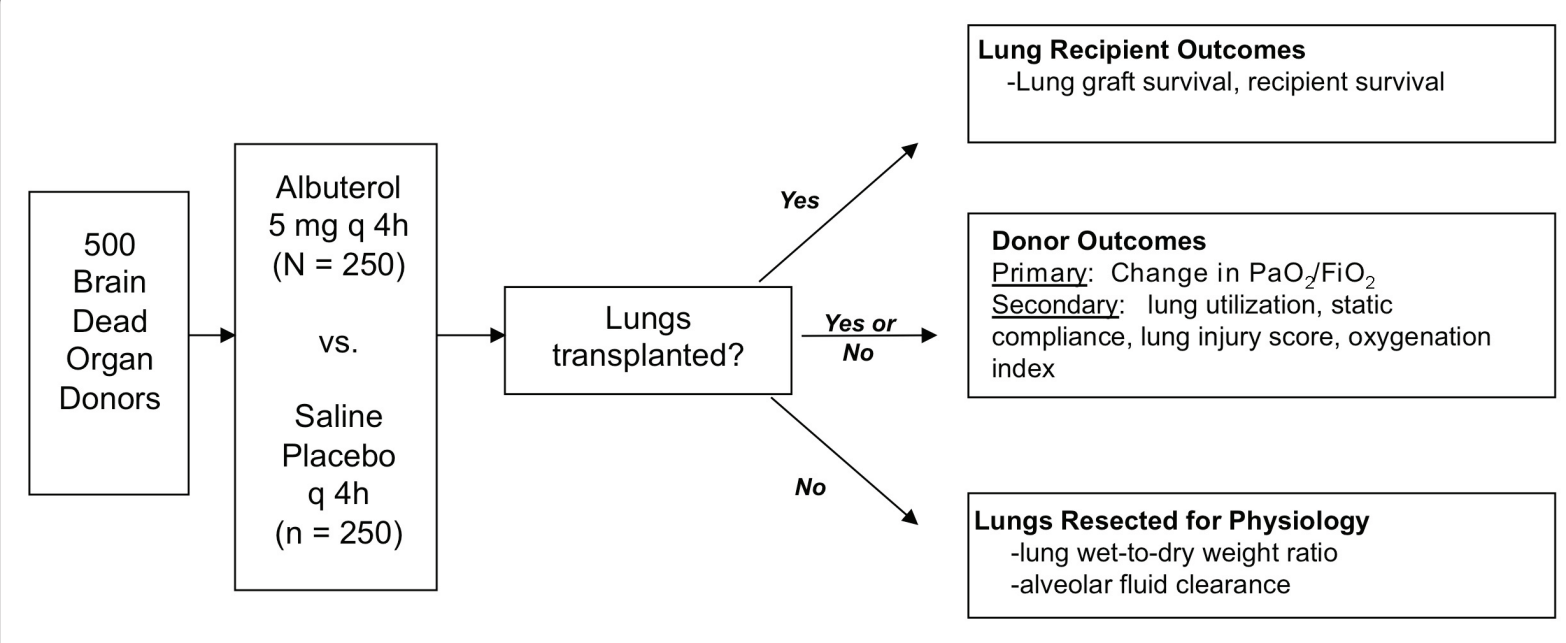
**Summary of clinical and physiological outcomes in the BOLD study**.

Study outcomes are summarized in Table 1 and Figure 2. The primary outcome is the change in donor oxygenation from enrollment to organ procurement. This is defined as the change in the PaO_2_/FiO_2 _ratio as measured by arterial blood gas analysis from enrollment to organ procurement or 72 hours, whichever occurs first. In addition to the overall change in PaO_2_/FiO_2_, the change in the area under the curve for all measurements of PaO_2_/FiO_2 _will be evaluated.

**Table 1:** Primary and secondary outcomes for the BOLD study

Type of outcome	Outcome	Definition
**Primary**	Donor oxygenation	Change in the PaO_2_/FiO_2 _ratio as measured by arterial blood gas analysis from enrollment to organ procurement or 72 hours, whichever occurs first
**Secondary clinical**	Donor lung utilization rate	The number of lungs transplanted divided by the total number of lungs available in the donors enrolled in each study arm. The donor utilization rate will also be evaluated using only potentially transplantable lungs in the denominator. For this analysis, donors whose lungs have absolute contraindications to transplantation will not excluded including donors with (1) significant pulmonary disease, (2) bilateral lung contusion, (3) hepatitis C antibody positive (4) age over 65 years or (5) HIV positive.
	Static lung compliance	Change in static lung compliance between enrollment and organ procurement or 72 hours, whichever occurs first. During study enrollment, static lung compliance is measured every 12 hours and immediately prior to organ procurement.
	Chest radiographic score	Change in chest radiographic pulmonary edema score between enrollment radiograph and radiograph obtained just prior to organ procurement. Chest radiographs are scored in a blinded fashion by two of the investigators using a scoring system developed and validated specifically for this study [29].
**Secondary physiological** (only in lungs that are not used for transplantation)	Lung wet-to-dry weight ratio	Gravimetric measurement of the ratio of the wet weight compared to the lung weight after drying. The lung wet-to-dry weight ratio is a quantitative index of the degree of pulmonary edema [17]. Total lung weight will also be measured.
	Rate of alveolar fluid clearance	The rate of alveolar fluid clearance is measured in a rewarmed lobe of the excised lung after instillation of an isotonic albumin-containing solution [17]
**Secondary recipient** (only in lung transplant recipients)	30-day lung graft survival	Percent of lung allografts that are functional 30 days after transplantation in each study arm
	30-day lung recipient survival	Percent of lung transplant recipients that are alive at 30 days after transplantation in each study arm

#### Secondary clinical outcomes

The effect of albuterol on several secondary clinical outcomes will be evaluated (Table 1), including the donor lung utilization rate, the change in static lung compliance from enrollment to organ procurement, and the change in chest radiographic score from enrollment to organ procurement.

##### Recipient outcomes

Lung and other solid organ recipient outcomes will be analyzed as secondary outcomes, including 30-day graft and recipient survival.

#### Secondary physiologic outcomes

Lungs that are not used for clinical transplantation are resected without perfusion and transported to the BOLD Lung Physiology Laboratory at UCSF for physiologic evaluation, including measurement of the lung wet-to-dry weight ratio and the rate of alveolar fluid clearance [17].

### Selection of study subjects

The inclusion and exclusion criteria are intended to maximize enrollment. We considered excluding donors in whom there is an absolute contraindication to lung transplantation (such as serious preexisting lung disease), because the secondary outcome of donor lung utilization could not be improved by albuterol treatment in this group. However, because the primary outcome (donor oxygenation) could theoretically be improved by albuterol in all donors, and contraindications to lung transplantation are not always apparent at the beginning of the donor management period, we chose to include all donors. All brain-dead organ donors managed by the CTDN who are 14 years of age or older and have next-of-kin consent for organ donation and research are eligible for enrollment in the clinical arm of the study. For inclusion in the secondary physiologic outcomes arm of the study, the lungs also must be rejected for transplantation and approved for research use by the coroner or medical examiner. For inclusion in the secondary physiologic outcomes arm of the study, a qualified surgeon must be available to resect the lungs at the time of organ procurement.

In addition to the above-mentioned inclusion criteria, for a donor to be included in the final analysis, they must receive at least one dose of study drug and complete the donation process as defined by surgical procurement of at least one organ. Five to ten percent of donors managed by the CTDN do not complete the organ donation process usually due to severe hemodynamic instability and/or multiorgan system failure. Because these donors are typically managed for less than 12 hours, they would be unlikely to receive more than one or two doses of the study drug and would have an inadequate time period over which to be assessed for the primary and secondary outcomes.

#### Consent process

The complex ethical issues that arise in donor management clinical trials were the subject of a recent review [19] and will not be considered in detail here. Under United States federal regulations, brain-dead organ donors are not legally considered to be human subjects for the purposes of informed consent. For this reason, the consent process for the BOLD study has been tailored for the donor population. At the time that a CTDN representative meets with family members of the deceased to obtain consent for organ donation, consent for research, including donor management studies, also is discussed. If the family member consents for research, then the donor becomes eligible for enrollment in the BOLD study.

Regarding consent from lung transplant recipients who will receive lungs from donors enrolled in the study, a panel of consulting transplant bioethicists have concurred that informed consent from lung recipients is not required in the BOLD study, because the study poses minimal risk, it has traditionally been the role of the transplant surgeon to determine the relative risk of an organ, and finally, there is no precedent in the transplant community for requiring recipient consent for donor management studies that pose minimal risk.

### Treatment groups and randomization

There are two treatment groups. The albuterol group receives 5.0 mg of albuterol by nebulization every 4 hours from the time of study enrollment until organ procurement. The placebo group receives an equivalent volume of nebulized saline every 4 hours.

Subjects are prospectively randomized in a 1:1 ratio among study and placebo groups. Randomization is conducted by the UCSF Investigational Pharmacy. The Investigational Pharmacy prepares study drug and placebo in identical vials that are randomly assigned study numbers in permuted blocks of eight. Sufficient study drug for one donor is placed in individual donor study kits that are distributed to the CTDN Transplant Coordinators for use when a donor is enrolled in the study. Each Transplant Coordinator maintains a stock of these kits so that a kit is always available when a Transplant Coordinator is on site at any of the 175 hospitals served by the CTDN. Each study subject is assigned a number that corresponds to the number on the study drug vial as part of the randomization process, and the number becomes that subject's unique treatment number.

Blinding of the study drug is preserved throughout the study. No treatment group information is provided to the investigators or CTDN staff except in case of an emergency, and a log of unblinding events is maintained.

### Study procedures

**Figure 3:**
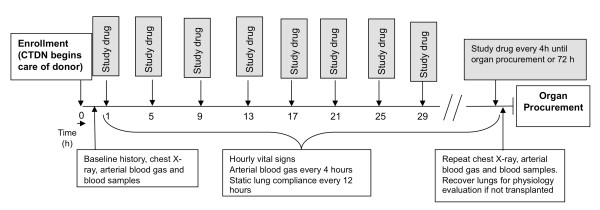
**Timeline for study procedures in the BOLD study**.

The study flow is summarized in Figure 3. All study procedures and primary data collection is performed by the CTDN Transplant Coordinators who are responsible for the clinical management of the organ donor onsite at the hospital. Enrollment into the BOLD study occurs once the CTDN assumes clinical care of the brain-dead patient with consent for organ donation and research and upon meeting inclusion and exclusion criteria. Nebulized study drug (albuterol or identical saline placebo) is administered every 4 hours by using a standard nebulizer device provided in the individual donor study kit for 72 hours or until the donor is sent to the operating room for organ procurement, whichever occurs first. Arterial blood gas, static lung compliance, and chest radiograph are obtained before the first dose of study drug and immediately before organ procurement for assessment of the primary and secondary study endpoints. Donors are managed using standard CTDN protocols [20] except for the administration of study drug and data collection, with one exception. It is recommended that all donors enrolled in the BOLD study be ventilated with 10 cc/kg of tidal volume based on predicted body weight to minimize ventilator-associated lung injury [21]. However, ventilator settings remain at the discretion of the Transplant Coordinator and supervising Advanced Practice Coordinator and may be altered depending on the clinical circumstances.

### Study variables

Comprehensive data are collected for each donor. At enrollment, demographics, medical history, cause of brain death, smoking, alcohol and drug use history, and hospital course before brain death are recorded. Throughout the study period, hemodynamic and ventilatory parameters are recorded hourly along with medication administration, fluid balance, and culture and test results. At organ procurement, organ disposition for all solid organs is recorded along with reasons for nonallocation.

In addition to clinical data, blood is collected for plasma and DNA at study enrollment. These blood samples are transported to an HLA typing laboratory at UCSF by courier along with donor samples for HLA typing. A second plasma sample is obtained at the time of organ procurement and is transported to the UCSF HLA laboratory by courier along with samples that are collected from the donor for tissue banking. The HLA laboratory processes all blood immediately to separate plasma, which is frozen at -80C in small aliquots and shipped to the coordinating center at Vanderbilt.

Hard or digital copies of enrollment and procurement chest radiographs are mailed to the coordinating center at Vanderbilt where they are scored by two investigators blinded to treatment arm assignment.

### Sample size

The sample size was estimated using oxygenation data obtained from the CTDN for donors managed by standard protocols. The targeted enrollment is 500 donors who complete the organ donation process, 250 treated with albuterol, and 250 treated with placebo. This sample size yields a power of 0.8 to detect an increase in the primary outcome, donor oxygenation, expressed as the mean difference of PaO_2_/FiO_2_, by 37.5 using a two-sided, two-sample t-test with a significance level of 0.05. The principal analysis will be intention-to-treat, based on randomization assignment among donors who complete the donation process and receive at least one dose of study drug. Interim analyses are planned at sample sizes of 100 and 300 for safety (see below) and efficacy. Early stopping rules for efficacy are based on the observed difference in PaO_2_/FiO_2 _ratio. Using stopping rules described in Jennison and Turnbull [22], the *p*-value thresholds are 7.4 × 10^-6^, 0.01, and 0.04 at the two interim and the final analyses, respectively.

### Data safety and monitoring

#### Safety considerations

In a large randomized trial of intravenous albuterol (salbutamol) in acute lung injury, the primary side effects were tachycardia and cardiac arrhythmias [23]. In the BOLD study, heart rate is monitored continuously. If heart rate increases by > 30 bpm during the study drug aerosolization, the aerosol is stopped. Because fluctuations in heart rate may be multifactorial, the subject is evaluated and a cause for tachycardia other than study drug is sought and if identified, treated. The next scheduled aerosolization of study drug is given at 5 mg of albuterol or placebo, and if the heart rate increases by > 30 bpm again, the aerosol is stopped and subsequent study drug doses are reduced to 2.5 mg (albuterol or placebo). A subsequent increase in heart rate > 30 bpm results in the study drug being held for another 4 hours and restarted at the 2.5-mg dose. Any subsequent increases in heart rate of > 30 bpm result in discontinuation of study drug for the duration of the study.

In subjects developing sustained atrial or ventricular arrhythmias, the study drug is discontinued and the event is reported as an adverse event. Eligible subjects with preexisting atrial fibrillation or multifocal atrial rhythms with a controlled ventricular response may participate in this trial. For enrolled subjects with preexisting atrial fibrillation or multifocal atrial rhythms, study drug is dosed and subsequently adjusted, held, or discontinued based on change in baseline heart rates as previously described. If subjects develop more than four new premature ventricular contractions (PVCs) per minute during aerosolization of the study drug, then the treatment is stopped for the remainder of the study.

Because several clinical studies show that inhaled beta-2 agonists do not result in significant alterations in blood pressure, we have not established specific guidelines for dose adjustments for blood pressure. Alterations in blood pressure may be related to other clinical events or pharmacologic interventions.

#### Data Safety and Monitoring Board

The Data Safety and Monitoring Board (DSMB) monitors donor safety, protocol adherence, and data quality. The DSMB receives reports of serious adverse events. Interim safety analyses are performed after 100 and 300 patients to detect unexpected changes in lung utilization.

Pearson's chi-square test will be computed to assess differences in lung utilization between treatment groups. Statistical significance will be judged based on the nominal 0.05 level. If a difference in treatment groups is determined, the DSMB will break the blind to assess the nature of the observed difference, as well as other factors that might differ between groups (e.g., age, smoking status). Because the study is minimal risk, there is no plan for early stopping for futility.

### Quality control

Because donors can be enrolled at any of the 175 hospitals served by the CTDN, the study activities are performed primarily by the transplant coordinators who are at the bedside from before enrollment until after organ procurement. CTDN transplant coordinators arrange for the study drug to be administered by the hospital respiratory therapist, draw blood samples, and record study data. Transplant coordinators are a heterogeneous group with different backgrounds (nursing, respiratory therapy, emergency medical services), training, and experience and typically have no formal training in human research. All CTDN transplant coordinators were trained extensively in the study procedures before the study launch and refresher training is done frequently. Study data are reviewed by the principal investigator as they accrue, and all protocol violations are immediately reported to the CTDN study coordinator who contacts the respective transplant coordinator to discuss the protocol violation and provide additional training.

### Study design and implementation challenges

Brain-dead donors are a unique patient population. Implementing a randomized, controlled trial in donors has led to many challenges, which are addressed below.

#### Ventilator management

Although a recent study suggests that lower tidal volumes are superior to higher tidal volumes in the management of the organ donor [24], standard protocols in use by the CTDN at the start of the BOLD study in 2007 dictated a tidal volume of at least 10 ml/kg of actual body weight and PEEP of at least 5 mmHg with further increases up to a maximum of 15 ml/kg mandated if PaO_2 _on FiO_2 _of 1.0 was < 500 and/or chest radiograph was not clear. In addition, it was recommended that tidal volumes should be set at the highest possible range while maintaining peak inspiratory pressures less than 30 cmH_2_O. This protocol was well established for optimizing cardiopulmonary function and was associated with lung utilization rates that were among the highest nationally. However, because of the known adverse effects of high tidal volume on lung inflammation [25,26] and alveolar fluid clearance [27], we were concerned that these high tidal volumes might cause sufficient lung injury in donors in the BOLD study to render the lungs unable to respond to beta-adrenergic agonist-mediated stimulation of alveolar fluid clearance.

Although we would have preferred to use the NHLBI ARDsNet protective ventilatory strategy of 6 ml/kg of tidal volume based on predicted body weight [28], this preference had to be weighed against the CTDN priority for optimizing inflation volumes to demonstrate good lung function to promote lung utilization for transplantation. A compromise was reached at 10 ml/kg of predicted body weight. This integration of predicted body weight into the setting of tidal volume necessitated a significant degree of staff reeducation.

#### Hospital Institutional Review Boards

Because brain-dead organ donors are not legally considered to be human subjects for the purposes of Institutional Review, we did not seek approval from the institutional review boards (IRBs) of the 175 hospitals where a brain-dead donor enrolled in BOLD might be cared for by the CTDN. The IRBs at the coordinating center (Vanderbilt) and at the BOLD Lung Physiology Laboratory site at UCSF both confirmed that the study does not involve human subjects. However, in the course of implementing the study, we have encountered questions from several hospital IRBs when they have become aware of the study, usually as a result of questions from hospital respiratory therapists who are asked to administer the study drug. By request, the study has been submitted and approved by several hospital IRBs. One hospital declined to participate, although no reason was given and the protocol had received IRB approval. Another declined to participate because of a hospital policy barring use of nebulized medications in mechanically ventilated patients.

#### Correct study drug dosing

Study drug is supplied in a concentrated form that must be diluted with saline for nebulization. On occasion, errors by the hospital respiratory therapist have led to a failure to dilute the study drug appropriately with subsequent administration of two to three times the prescribed dose. To prevent this error, the BOLD study drug vial label was redesigned to highlight the dilution instructions. In addition, new procedures were put in place to mandate face-to-face communication between the transplant coordinator and the hospital respiratory therapist before study initiation. This issue highlights the challenge of implementing a clinical trial of a therapy that must be administered by a respiratory therapist to a donor whose care is managed by a transplant coordinator but who is still an inpatient at one of 175 disparate hospitals.

#### Management of bronchospasm

Although bronchospasm is not a common problem in organ donors, it does occur. To avoid reflexive open label use of albuterol for any symptoms of bronchospasm, a protocol for management of bronchospasm was put into place in the BOLD study such that decisions for management of bronchospasm are made in consultation with an Advanced Practice Coordinator. Use of alternative agents, such as ipratropium bromide, is encouraged but not protocolized and use of any open-label albuterol is recorded and requires a written explanation.

#### Protocol violations

The most common protocol violation has been failure to obtain baseline study variables, such as arterial blood gas, before study drug initiation. The transplant coordinator has the difficult task of simultaneously optimizing donor hemodynamics and organ function, arranging for a variety of ancillary tests, assisting grieving families, and assisting with organ allocation. The added work burden of conducting a research study within the short period of donor management can be challenging and may not take precedence during very busy times. Designing an intervention and case report from that could be seamlessly integrated into the usual flow of donor management was a challenge and ongoing reinforcement of the need to obtain baseline study variables has been required.

## Conclusions

To increase donor organ utilization, new donor management therapies are needed to optimize donor organ function. A randomized, clinical trial is the most reliable method to test different donor management therapies. Experience gained in the design and implementation of the BOLD trial illustrates some of the challenges inherent in donor research but also demonstrates that large, randomized, clinical trials are feasible. It is our hope that experience gained in the BOLD study will stimulate other investigators to test donor interventions in randomized, clinical trials in the donor population.

## Competing interests

The authors declare that they have no competing interests.

## Authors' contributions

LBW and MM conceived of the study and participated in its design and coordination and drafted the manuscript. TK and DB designed and implemented the analytic plan for the study. ML and EJ designed and implemented the study at the CTDN. SB oversaw all aspects of sample collection and processing for the study. GB provided input into the study design for ethical and IRB issues. All authors read and approved the final manuscript.
